# Beyond Charles Bonnet Syndrome: A Scoping Review of Psychotic Symptoms in Vision Loss

**DOI:** 10.31083/AP47195

**Published:** 2026-04-21

**Authors:** Georgios D. Floros, Petros Petrou, Ioanna Mylona

**Affiliations:** ^1^2nd Department of Psychiatry, Aristotle University of Thessaloniki, 541 24 Thessaloniki, Greece; ^2^First Department of Ophthalmology, General Hospital “G. Gennimatas”, National and Kapodistrian University of Athens, 115 27 Athens, Greece; ^3^Department of Ophthalmology, General Hospital of Serres, 621 00 Serres, Greece

**Keywords:** psychotic disorders, hallucinations, eye diseases, vision disorders

## Abstract

Despite ongoing discussions regarding the connection between overt psychosis and the impairment of visual function, the discourse primarily centers on the potential neurobiological association between major psychotic disorders and congenital blindness. Specific neurological lesions are associated with psychotic symptoms, including visual hallucinations. Defining and assessing psychotic-like symptoms remains problematic and the negative effects of vision loss on the development of psychotic-like symptoms have not been adequately studied. In this scoping review, we present a clear picture of this complex relationship, with conclusions that will assist the differential diagnosis of psychotic symptoms in the ophthalmological patient. A literature search was undertaken on primary studies and reviews about psychotic symptoms in eye disease outside the context of the Charles Bonnet syndrome and established dementia. Seven reviews and nine primary studies or case series were included in the article. Nearly all reviews and two primary studies centered on visual hallucinations (VH) and only three primary studies expanded the scope to include other psychotic symptoms. There are considerable gaps in our knowledge concerning the characteristics of psychotic symptoms in patients with eye disease, primarily due to the lack of willingness to examine the possibility that a significant number of psychotic symptoms other than VH exist. Furthermore, the lack of a consensus on a set of criteria for Charles Bonnet syndrome is hampering research that would assist clinical management and the field, overall. A shift in the field of psychiatric research to the study of other psychotic symptoms, including non-hallucinatory changes to typical visual perception, has been noted but remains in the very early stages. Building on this new knowledge base with the addition of psychotic symptoms in newly established visual disorders, will help both fields.

## Main Points

1. Psychotic symptoms associated with vision loss have been reported for some time, and following the formulation of the Charles Bonnet syndrome, the focus has been on visual hallucinations much to the detriment of reporting other symptoms that may or may not co-exist with visual hallucinations.

2. This scoping review found an extremely small number of primary studies that even examined the existence of other psychotic symptoms outside the context of dementia or pre-existing psychotic disorders—likewise reviews on the subject remain limited to examining visual hallucinations, without providing a convincing framework on their etiology.

3. More research focus is needed in the complete psychiatric assessment of patients who demonstrate visual hallucinations in the context of vision loss, but also, more population studies that include questions on broader psychotic symptomatology are also essential.

## 1. Introduction

### 1.1 Visual Function Deprivation and Psychotic Symptoms

Despite rising research interest regarding the connection between the deterioration of visual function and psychosis, the focus of this discourse primarily lies in determining whether significant psychotic disorders may have a neurobiological correlation with congenital blindness [1]. Sensory deprivation, particularly visual, has historically been linked to psychotic manifestations in individuals who did not previously report any [2, 3]. These symptoms can emerge rapidly following a brief period of sensory deprivation, with total visual deprivation resulting in hallucinations within just five consecutive days [3]. Sensory deprivation leads to visual hallucinations (VH), auditory hallucinations and paranoid ideation.

Our understanding of the association of visual function deprivation and psychotic symptoms mostly relates to VH. VH with preserved insight as to their nature are observed in Charles Bonnet Syndrome (CBS), a condition where individuals with significant visual impairment experience intricate VH, typically while retaining awareness of their true nature [4]. CBS is classified in the most recent eleventh version of the International Classification of Diseases (ICD-11), by the World Health Organization, under ‘9D56 Visual release hallucinations’ as the experience of complex exclusively VH in a person who has experienced partial or complete loss of vision, with those VH unrelated to mental or behavioral disorders. A comprehensive review of CBS found the collective prevalence across the life span to be 10.2% [5]; low vision, retinal diseases or glaucoma had similar rates, while being female and of higher age were associated with a higher prevalence of CBS. CBS is often underreported, primarily due to patients’ concerns about being misdiagnosed with a psychiatric condition, as well as a lack of awareness among healthcare professionals and patients alike [6]. Although it has been regarded as a benign and mostly self-limited phenomenon, the underlying mechanisms remain incompletely understood. Recent studies have suggested a potential role of cortical disinhibition and compensatory neural activity in the visual cortex as a spontaneous response to loss of input [7, 8]. It has long been confirmed that ‘true’ CBS does not present with other psychiatric symptom comorbidity and is self-limited [9], although a study of 492 patients reviewed found that CBS is of longer duration than previously suspected while a third of all patients had significant complaints [10].

Visual hallucinations are typically singled-out as the most common psychotic symptoms in visual impairment, however, there are multiple possible causes other than CBS. A recent meta-analysis of fourteen studies on primary and secondary psychosis patients with VH found those significantly associated with secondary psychosis—here the type of secondary psychosis (either organic, drug-induced or mixed) was non-significant. Interestingly, hallucinations of inanimate objects were significantly more likely to be associated with secondary psychosis [11]. Lewy-body dementia stands out as the most likely cause of complex VH in the elderly [12], and this may precede the onset of severe cognitive impairment [13], the course of the disease is dependent on the underlying pathology and unrelated to vision status. An often overlooked cause of VH in the absence of prior psychiatric history is cases of CBS related to neurosurgery, evident either pre- or post-surgical procedures [14]. Differential diagnosis may be complicated if the symptoms arise in the absence of other symptoms related to the underlying cause, such as seizures or optic nerve involvement.

There are numerous confounding factors that might contribute to both vision impairment and psychiatric symptoms other than visual impairment or specific neuropathology. Loneliness has been confirmed as a risk factor for depressive and psychotic symptoms both in the general population [15] and in adults with dementia [16]. Impaired cognition in old age is a well-known factor in the appearance of psychotic symptoms; the differential diagnosis of these symptoms, in the absence of a psychotic history, cognitive impairment, or a specific neuropsychiatric syndrome, may be linked to neurodegenerative processes that precede dementia [17]. Dementia-related psychosis is relatively prevalent, as approximately half a million cases were recorded in Medicare claims from 2013 to 2018 in the United States of America, out of a total of 2.5 million patients experiencing psychosis. With vision gradually attenuating with old age in parallel with cognition, a reciprocal relationship has been noted, with vision loss identified as a potentially modifiable risk factor for dementia [18].

### 1.2 Discerning Between Different Types of Psychotic Symptoms

Psychotic symptoms are generally characterized by significant disruptions in perception, thought processes, and behavior, often indicated by a disconnection from reality. These symptoms may encompass a range of hallucinations, delusions, disorganized thinking or speech, and severely disorganized or catatonic behavior. Typically, patients exhibit a lack of awareness concerning the actual nature of their symptoms. Research indicates that the prevalence of any psychotic symptom within a large non-demented population can reach as high as 10.1% [19], and these were linked to poor prognosis. There has been a recent development in the grouping of associated symptoms into a category of ‘psychotic-like’ symptoms [20]. A crucial element in the classification of psychotic-like symptoms is the differentiation between transient perceptual disturbances, prodromal symptoms of psychosis, and overt psychotic symptoms. Transient perceptual disturbances (TPD) are generally brief, minor changes in perception (visual, auditory, tactile, etc.) that do not signify a disconnection from reality and are frequently comprehensible within a specific context, particularly as a reaction to fatigue, stress, substance use, sensory deprivation, or lack of sleep [21]. These experiences are typically brief, seldom exceeding a few minutes, and are self-limiting. Insight is generally maintained: the individual may acknowledge the experience as atypical or unreal, and it is not linked to disorganized thought processes or behaviors. Common examples in this category include hypnagogic or hypnopompic hallucinations, such as the sensation of hearing one’s name called in a silent room. Complex perceptual disturbances, particularly in social perception, are usually ascribed to neurological impairments (like traumatic brain injury) and are therefore inherently non-transitory [21]. Psychotic experiences (PE) are characterized as hallucinations or delusions that may manifest in individuals who do not have a psychotic disorder within the general population; they serve as an indicator of significant psychopathology and a transdiagnostic sign for the potential onset of mental disorders [22]. In individuals aged over 64, for every 100 older adults, 1 individual reports an incident of PEs annually. In contrast, among adolescents, this figure increases from one to five, significantly influenced by the higher usage of psychoactive substances within this demographic. Non-hallucinatory changes to everyday visual perceptual experience are symptoms that do not constitute a hallucinatory phenomenon but closely resemble it. Those symptoms included in a related detection scale, the Bonn Scale for the Assessment of Basic Symptoms [23] are: unclear sight-phasic, momentary blindness, partial vision, sensitivity to light, photopsia, near sight, changes in size, changes in form, changes in color, changes in others’ face or body, mirror-related phenomena, movements of objects experienced as related to own movements, diplopia, oblique vision, disturbances in estimation of distances or size, disintegration in perception of linearity of contours, abnormally long-lasting retinal after-image. Basic symptoms can predict progression to full-blown psychosis from the state of being at high-risk for psychosis [24]. A series of visual tests has been proposed as a more objective battery for ascertaining risk for transcending the boundary of at-risk status to full-blown psychosis, including tests of visual thalamocortical hyperconnectivity, decreased visually evoked occipital P1 amplitudes in electroencephalogram and reduced occipital gamma band power during visual detection in magnetoencephalography. Other possible predictive retinal indicators consist of reduced cone a- and b-wave amplitudes, as well as a weakened photopic flicker response observed during electroretinography [25].

### 1.3 Rationale of This Review

Whereas CBS is widely accepted as a possibility when VH occurs in previously unaffected individuals with recent vision loss, the case for the induction of psychotic or psychotic-like symptoms and experiences (such as paranoid thoughts and perceptual disturbances), with the potential for either full or partial recovery, remains unclear. The purpose of this review is to critically assess the theoretical framework and primary research on psychotic symptoms outside the typical CBS diagnosis (including only VH with insight).

### 1.4 Objective of This Review

The literature review attempted to ascertain whether there are relevant studies or case reports/case series that would help in answering the following questions:

a. How is vision loss linked to the appearance of psychotic and psychotic-like symptoms, outside of well-known syndromes (CBS, dementias)?

b. How can we differentiate between transient psychotic, psychotic and psychotic-like symptoms in the ophthalmological patient?

c. A secondary research question was when do psychotic-like symptoms in the ophthalmological patient merit psychiatric treatment?

## 2. Materials and Methods

**Fig. 1.**
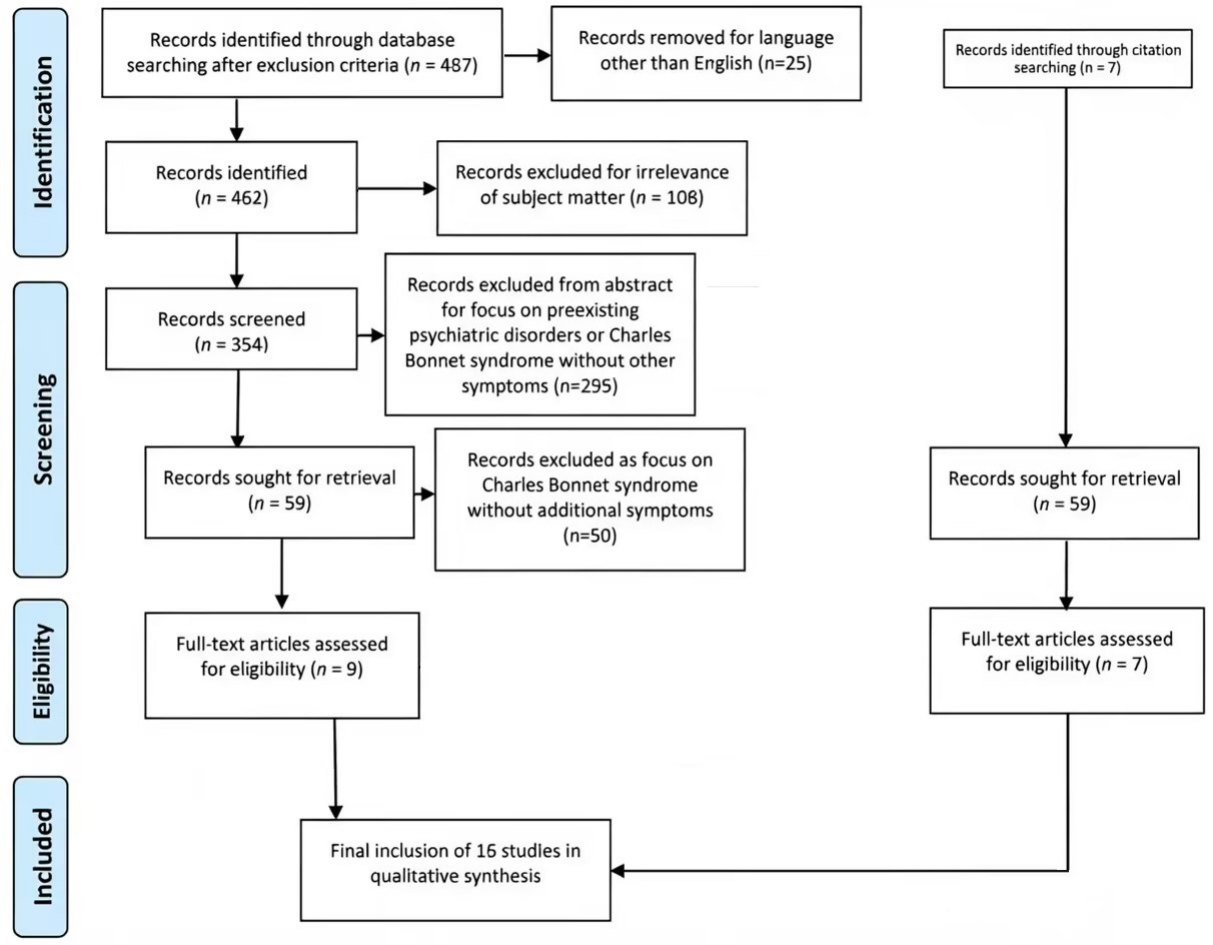
**This presents the flowchart for the selection of studies that were included**.

The review follows Preferred Reporting Items for Systematic reviews and Meta-Analyses (PRISMA) guidelines for a scoping review and the full protocol for the review is registered with the Open Science Framework (OSF) and published at https://doi.org/10.17605/OSF.IO/CKXSB. The associated PRISMA checklist is provided as **Supplementary Material**. Literature review spanned PubMed (https://pubmed.ncbi.nlm.nih.gov/), Web of Science (https://www.webofscience.com/) and Scopus (https://www.scopus.com/) with an appropriate string (psychotic OR psychosis OR psychotic-like OR delusions) AND (ophthalmology OR eye disorder OR ophthalmic OR cataract OR glaucoma OR age-related macular degeneration OR retinopathy). Search excluded articles older than forty years and with no full text available in other languages than English. Inclusion criteria: Adult population with an ophthalmological condition. Exclusion criteria: Adolescent population, patients with pre-existing serious mental disorder (including any psychotic disorder, bipolar disorder, schizoaffective disorder, major depression with psychotic features, active substance abuse, schizoid or schizotypal personality disorder). Google Scholar (https://scholar.google.com/) was employed to locate grey literature that includes conference proceedings and abstracts along with dissertations and theses. Both primary studies and reviews were included in the search. The search was performed independently by two researchers (G.F and I.M) with discrepancies resolved by a third researcher (P.P). Data were summarized by all researchers following consultation and consensus. The flowchart for the selection process is provided in Fig. 1.

## 3. Results

Out of 354 potential articles screened from the search engines only nine met the inclusion and exclusion criteria; two were reviews on visual hallucinations only [26, 27], five were clinical studies [28, 29, 30, 31, 32] and two were presenting a case report or series [33, 34]. Additional studies that were included through references of those studies included five more reviews [35, 36, 37, 38, 39], a retrospective cohort study [40] and a cross-sectional study [41]. Review studies are presented in Table 1 (Ref. [26, 27, 35, 36, 37, 38, 39]) while articles detailing primary research and case reports or series are presented in Table 2 (Ref. [28, 29, 30, 31, 32, 33, 34, 40, 41]).

**Table 1.** **Review studies**.

Study	Remarks
Pelak and Liu [27]	A brief outline of causes for VH^1^ including cases in ophthalmological patients, focusing on treatment options.
O’Brien *et al*. [26]	Comprehensive review of VH in neurological and ophthalmological patients.
Ffytche [35]	Comprehensive review of VH in eye disease but does not mention any accompanying signs or symptoms of psychosis.
Bernardin *et al*. [37]	Review of abnormalities of the retina and their potential link with the occurrence of VH in eye disease, neurological disorders and psychosis.
Stoltzner and Duncan [36]	Limited review on all possible causes of VH, including eye disease.
Waters *et al*. [38]	Comprehensive review of VH in various psychotic disorders, eye disorders, neurodegenerative disorders and general population tackling epidemiology, phenomenology, assessment and treatment options.
Shoham *et al*. [39]	Review of studies linking psychosis and visual acuity in various clinical settings.

^1^ VH, visual hallucinations.

**Table 2.** **Primary research studies and case reports/series**.

Study	Type	Assessment tool	Main findings
Montagnese *et al*. [28]	Cross-sectional, between-group comparison	Mini Mental State Exam (MMSE), North East Visual Hallucination Interview (NEVHI)	Distress, cognition and hallucination-specific insight are linked similarly across neurological disorders and eye disease. No data on visual acuity or exact eyesight issues were recorded directly.
Shoham *et al*. [30]	Cross-sectional, within group comparison	Psychosis Screening Questionnaire (PSQ), Social Functioning Questionnaire	Visual impairment correlated with positive results in the PSQ scale which is suitable for identifying psychosis in general practice. No data on visual acuity or exact eyesight issues were recorded directly.
Dudley *et al*. [29]	Cross-sectional, between-group comparison	MMSE, NEVHI, visual acuity, verbal fluency test, category fluency test, Unified Parkinson’s disease rating scale (UPDRS) part II and part III, Epworth Sleepiness scale, Mayo sleep questionnaire	Single modality hallucinations varied in prevalence across disorders with eye disease patients reporting fewer multimodal hallucinations with mostly visual and tactile hallucinations.
Subramaniam *et al*. [41]	Cross-sectional, within group comparison	Geriatric Mental State examination (GMS), Community Screening Instrument for Dementia (CSI’D), World Health Organization disability assessment schedule (WHO-DAS II), Client Service Receipt Inventory (CSRI)	In non-demented individuals with eyesight problems there were statistically significant odds for VH but not for paranoid ideation or persecutory delusions. No data on visual acuity or exact eyesight issues were recorded directly.
Kinoshita *et al*. [40]	Population survey	World Health Organization Composite International Diagnostic Interview (CIDI) 3.0	The association between visual impairments and hallucinations was significant in those aged >60, whereas to auditory hallucinations in those aged <39. All data on visual acuity were from self-report.
Serlin *et al*. [32]	Retrospective case-control	Neuropsychiatric outcomes from patient files, visual data from comprehensive clinical examination	Group with proliferative retinopathy had higher chance of a new diagnosis of unspecified psychosis compared to non-proliferative retinopathy.
Floros *et al*. [31]	Longitudinal cohort	Visual acuity, Symptoms Checklist 90 - revised	Patients with severe vision loss showed a reduction of paranoid ideation and psychoticism symptoms following cataract surgery and partial restoration of vision within a two-month span.
Casey and Wandzilak [33]	Case series	Clinical examination	Visual hallucinations with associated delusional content and no insight in two cases of advanced senile macular degeneration.
Hasoglu *et al*. [34]	Case report	Clinical examination	Single case of a 26-year-old man who developed frank psychosis without visual hallucinations following acquired partial blindness.

### 3.1 Review Studies 

It is striking that nearly all reviews were limited to VH only (Table 1). While there was a useful comparison across various disorders in a number of articles [26, 27, 36, 38], discussion typically did not expand to any accompanying signs or symptoms, seemingly negating the possibility that VH could be just one in a series of symptoms. There is an expansion of the scope in a single review [26], where the authors include the possibility of multimodality hallucinations (VH combined with other types of hallucinations) and secondary delusions (delusions related to the content of the VH and added as explanatory afterthoughts). Nevertheless, the authors of the study only mention Charles Bonnet syndrome as the sole possible instance of VH in eye disease, despite the absence of multimodal hallucinations being a typical inclusion criterion for Charles Bonnet syndrome. Even within the context of treatment for visual hallucinations, the options that are presented in all instances [26, 27, 36, 38] are poorly researched—there is a notable lack of quantitative data regarding the various treatment modalities that are presented; the authors in all instances quote a small number of clinical studies or even case series when suggesting possible treatment. While antipsychotics are cautiously suggested as possible treatment, there is no working hypothesis as to their neurobiological suitability based on pharmacokinetics and class properties (e.g., receptor binding and brain pathways that are implicated). The most useful review in this respect is the work by Waters *et al*. [38], comparing VH across the psychotic spectrum, eye disease, neurodegenerative disease and the general population. The authors make the interesting point that VH are understudied even in psychiatric literature. They assess that VH in psychosis has remarkable similarities to auditory hallucinations and to VH in eye disease regarding clarity, frequency, form and character but differ in that the latter carries a smaller emotional burden, lack of sense of personal significance and higher frequency of simple images or patterns. Patients are typically relieved when they are informed that the occurrence is relatively common in eye disease in contrast to the lack of insight in psychosis where stress persists since the patient will not alter their viewpoint. The authors noted the lack of studies directly comparing VH across disorders and included a table of reported frequency of <10% for multimodal hallucinations and delusions, which co-exist with VH from a referenced study [42]. A useful algorithm is offered for the assessment of VH with certain exclusion items (dreams, imagery, eye floaters, etc.).

The single review that expanded the scope to any psychotic symptoms is an all-encompassing systematic review of studies that associate visual acuity and psychosis in any clinical or non-clinical setting [39]. The authors concluded that a cross-sectional association between visual acuity impairment and psychosis was supported by the evidence, however most relevant studies related to distant future outcomes in young individuals (e.g., conscripts developing a psychotic disorder in the distant future) or senior patients with varying levels of cognitive decline. Furthermore, there was little mention of the nature of psychotic symptoms that were reported and no effort to discern them, rendering this review of limited help in the current research questions.

### 3.2 Primary Research and Case Reports/Series 

There were very few high-quality studies that actively sought out to ascertain the existence of psychotic symptoms other than visual hallucinations in patients with eye disease, with two studies focusing on visual hallucinations: a study of 82 persons with various eye diseases where visual hallucinations were present found there was a small chance (3.7%) that other hallucinations, almost exclusively tactile, may co-exist [29]. Unfortunately, there was no provision for detecting any other type of psychotic symptoms. A general population survey by Kinoshita *et al*. [40] found that the association between visual impairments and hallucinations was statistically significant only in those persons aged >60, whereas the association between visual impairments and auditory hallucinations was statistically significant only in those aged <39. This finding could be related to a relationship between cognition impairment and visual hallucinations, which remains robust across not just neurodegenerative disorders [41], such as Parkinson’s disease or Lewy body dementia, but also eye disease, as evidenced in a comparative study [28]; insight in particular was linked to higher cognitive scores and less severity and distress in all groups.

There were only three studies that assessed a wide gamut of psychotic symptoms in patients with eye disease: Floros *et al*. [31] compared 200 consecutive cataract patients, with severe vision loss, on symptoms of Paranoid Ideation and Psychoticism pre and post cataract surgery. Those symptoms were assessed with the Symptoms Checklist 90 - revised (SCL-90-R) scale. Results showed cataract surgery associated with a reduction in those symptoms, while higher improvement was associated with higher improvement in visual acuity while controlling for age, gender and stressful life events during the past six months. Notably, items on the two subscales (Paranoid Ideation and Psychoticism) do not assess the presence of visual hallucinations. Items on Paranoid Ideation referred to paranoid readiness and assignment of blame while items on Psychoticism related to auditory hallucinations and various types of delusions. The most inclusive study is the one by Shoham *et al*. [30] who detailed results from the United Kingdom Adult Psychiatric Morbidity Survey carried out on 2014 with 7546 participants. The researchers employed the Psychosis Screening Questionnaire (PSQ) to detect psychotic symptoms. It contained questions on five clusters of psychotic symptoms, including thought interference, persecution, perceptual abnormalities, strange experiences and hallucinosis. Unfortunately, there was no standardized assessment of visual impairment, with the authors relying on ad hoc questions. Social functioning assessment was also carried out, to assess its role as a potential mediator. There was a positive association between visual impairment and psychotic symptoms, even after controlling for potential confounders, while mediation analysis suggested that reduced social functioning accounted for 42% of the association between any hearing impairment and positive PSQ result. Excluding those who screened positive on the PSQ solely due to reporting hallucinations did not significantly affect those associations, but visual hallucinations were not associated with hearing impairment. Age and sensory impairment did not have an interaction in influencing the PSQ result. Another study by Subramaniam *et al*. [41] assessed 2565 elderly non-demented individuals for hallucinations, persecutory delusions and paranoid ideation. The authors found that those individuals with eyesight problems had higher odds for VH but not for paranoid ideation or persecutory delusions. However, the latter conclusion was only confirmed with limited items on a semi-structured interview and eyesight issues were elicited from a functional assessment and not an ophthalmic examination or visual acuity data.

## 4. Discussion

### 4.1 Visual Hallucinations in Visual Defects—A First Step Towards Psychosis?

Review of the literature showed considerable gaps in our knowledge base regarding the nature of psychotic symptoms in patients with eye disease, even regarding visual hallucinations, whose presence has long been acknowledged. The reviews that are referenced here are mostly centered around the less complicated cases of Charles Bonnet syndrome, offering descriptive phenomenology, epidemiology and treatment options; however, the lack of a unifying framework renders treatment suggestions symptomatic and of doubtful clinical value. This is indirectly inferred by the complete lack of any larger scale clinical studies on treatment. VH remain an under researched topic both in psychiatry and in other disciplines due to the periodic nature of their appearance, the stigma associated with their presence and a lack of scientific rigor in their observation up to relatively recently [42]. Their diverse phenomenology and different underlying pathophysiological mechanisms are indirectly evident when we examine even the few studies that are referenced here. There are, in fact, numerous proposed mechanisms that may play a role in VH in eye disease and an attempt to present a general framework by Collerton *et al*. [43] centered around the lack of sensory data to resolve prediction errors that arise from expectancies that are driven by context, emotion, intention, motivation and memories. Each of those factors, however, may also play an important role in the appearance of other psychotic symptoms. It has recently been suggested that visual experience plays a vital role in the development of our internal world model, and that the functionality of this model may be significantly impaired after experiencing visual loss [44]. The authors have presented a computational model that elucidates how congenital visual impairment serves as a protective factor against psychosis, whereas visual impairment occurring later in life increases susceptibility to it. This model includes several hypotheses that require further testing in individuals who are congenitally blind. It is presumed that congenitally blind individuals are shielded from psychosis due to alterations in N-methyl-D-aspartate (NMDA) receptor structure induced by visual loss, which occur over a sufficiently extended developmental timeframe. In contrast, visual impairment experienced later in life is unable to activate the same protective mechanisms, as brain function has already been established. Instead, the loss of visual function, especially if it is combined with defects in cognition common to old age, negative emotional response to the loss of visual function and reduced motivation due to those changes and/or social isolation would serve to exacerbate all psychotic phenomena. A recent study of patients with psychosis attempted to ascertain which brain areas are activated during VH. Although the study only included six individuals, in all instances the primary visual cortex was either inactive or under-activated, supporting the notion of a separation of advanced visual processing regions from the primary visual cortex shifts conscious perception away from reality and towards internally produced perceptions [45]. This hypothesis is compatible with the appearance of VH in eye disorders since the primary visual cortex is effectively inactive due to reduced input.

The primary research studies that are included in this review are very few, denoting the lack of attention to this issue despite the first case series where psychosis developed following eye surgery dating back to 1935 [46]. It appears that the introduction of the Charles Bonnet syndrome during the 1930s has coined a term which conveniently absorbed nearly all cases with psychotic symptoms, shifting attention to VH; this has not however helped produce advances in research with practically no large clinical studies regarding treatment of Charles Bonnet syndrome, which still does not have clear inclusion and exclusion criteria in either the ICD-11 or the Diagnostics and Statistical Manual of Psychiatric Disorders (as compiled by the American Psychiatric Association). In fact, to date there persists the contradiction of defining Charles Bonnet syndrome as strictly including VH with insight while mentioning the possibility of multimodal hallucinations or secondary delusions [26].

Methodological issues were also evident in most of the referenced primary research studies (Table 2), with no data on visual acuity or exact eyesight issues recorded in four studies [28, 30, 40, 41], while five studies did not include a full assessment of psychic status [28, 29, 30, 31, 41]. Research design that favors highly specific scales, such as the North East Visual Hallucination Interview (NEVHI), increases specificity to the symptoms that were sought after (for example, hallucinations only with the NEVHI) but assumes less significance to the presence of other psychotic symptoms.

It is worth noting that a reverse bias against studying VH in general and focusing on other psychotic symptoms is active in psychiatry; VH tend to be attributed more readily to neurological disorders compared to auditory hallucinations despite the fact that their absolute frequency remains high in psychosis; a recent clinical study of 1119 patients with non-affective psychosis found a lifetime prevalence of 37% for VH and frequent co-occurrence with auditory hallucinations. This incidence is in fact higher than the comparative incidence for VH in dementia with Lewy bodies and similar to the incidence in Parkinson’s disease. Still, there is also a lack of studies with modern imaging techniques in psychotic patients with VH, save for the aforementioned study [45].

While concluding this review, it is important to note the importance of cross-disciplinary cooperation with otorhinolaryngology specialists in cases of auditory and olfactory hallucinations. A study of 1007 subjects aged 18–92, who were referred for audiometric testing, found that 16% of individuals with hearing impairment had experienced auditory hallucinations, including hearing voices, with this incidence raised to 24% in individuals with severe hearing impairment [47]. Qualitative alterations or distortions in olfaction (dysosmias) may be more upsetting to a patient than the complete loss of function and include distorted quality of an odorant stimulation as in troposmia and the perception of an odor when no odorant is present as in phantosmia or hallucination [48]. While the detailed presentation of those cases is outside the scope of this review, a presentation of predominantly auditory or olfactory symptoms warrants a detailed otorhinolaryngological examination.

### 4.2 Limitations of This Review Process

A general limitation of the review process relates to the underdevelopment of the field; there were extremely few studies that directly assessed all types of psychotic symptoms in patients with vision loss and the useful material in most referenced studies was like a footnote compared to the overwhelming body of evidence on VH. It is thus unclear whether similar material remains underreported in general. The lack of consensus for CBS also made the review process harder since every case that was noted as such had to be reviewed for the presence of psychotic symptoms other than VH.

### 4.3 Research Obstacles and Practical Solutions in the Study of Psychotic Symptoms in Eye Disease

The current body of knowledge is scarce and does not provide convincing answers to our primary and secondary research questions. The principal research obstacle appears to be the lack of willingness to examine the possibility that other psychotic symptoms than VH do exist, regardless of their smaller comparative incidence to VH; the studies which did research the existence of other psychotic signs or symptoms reveal that these are not all that uncommon [31, 32, 40], although more uncommon than VH [29], especially in patients of old age [41]. The existence of those symptoms, albeit in reduced frequency compared to VH, points to the possibility of dual mechanisms of appearance with local (sensory) mechanisms producing a larger percentage of VH in eye disease as suggested earlier [49] while the lack of corrective input regarding the surroundings would boost other psychotic symptoms. Also, VH without insight and multimodal hallucinations could have a mixed etiology combining sensory and top-down factors. Major moderating factors are cognitive and emotional status. Younger patients tend to present more frequently with auditory hallucinations; it is unclear as to whether this can be attributed to better cognitive status filtering out more easily deficits in visual processing.

Examining psychotic symptoms in depth is typically out of the scope of the clinical history that the patient’s ophthalmologist is trained to take. Furthermore, there is a bias against reporting any psychotic symptoms as a patient, so as not to be considered as ‘crazy’, and thus those symptoms need to be elicited. Self-report questionnaires are of limited practical use when there is a considerable visual defect. A helpful intermediary would be a specialized nurse who could devote more time to a semi-structured interview with the patient and, if necessary, the caregiver. A psychiatrist or clinical psychologist should be consulted when symptoms diverge from the typical clinical picture for CBS in that there is reduced insight for any VH and/or other psychotic symptoms exist. Conversely, the specialized tests that are detailed in a review of ophthalmological basic symptoms [24] necessitate the inclusion of ophthalmologists in the research teams examining the transition to psychosis from at-risk status.

### 4.4 Directions for Research

Research in the domain would benefit greatly from a common set of criteria for Charles Bonnet syndrome that would limit its applicability to individual cases with clear presentation including only VH with clear insight and absence of anosognosia (but inquiring about the full gamut of psychotic symptoms) and a full cognitive and emotional status examination. Currently, this syndrome is outside the established disorder nomenclature for psychiatric (DSM-5) or any other type of disorder (ICD-11). Comparative studies between patients with eye disease and psychosis, including examination with modern imaging techniques during VH are necessary to ascertain the neurobiological commonalities and differences between the phases of transition to psychotic symptoms among different populations, shedding light to those complex interactions. Extending the scope of ophthalmological tests that are proposed for people at high-risk for psychosis and basic symptoms to include patients with more pronounced visual function loss and no prior psychiatric history, could provide valuable comparative data. This process would, for example, include validating scales as the “Bonn Scale for the Assessment of Basic Symptoms” in populations with visual impairment. Multidisciplinary research teams would include specialists from ophthalmology, psychiatry, neuroscience and otorhinolaryngology. Population studies that assess subjects with vision should detail the full range of psychotic symptoms and not just VH.

## 5. Conclusions

There is a dearth of primary studies on the wide range of psychotic symptoms and the impairment of visual function, with the focus almost exclusively centered on visual hallucinations. Expanding the scope of research to include the study of other psychotic symptoms, including non-hallucinatory changes to typical visual perception, is necessary to further our understanding of the complex relationship between sensory perception and higher cognitive functioning.

## Supplementary Material

Supplementary material associated with this article can be found, in the online version, at https://doi.org/10.31083/AP47195.
